# Sporotrichosis in Sub-Himalayan India

**DOI:** 10.1371/journal.pntd.0001673

**Published:** 2012-06-12

**Authors:** Santwana Verma, Ghanshyam K. Verma, Gagandeep Singh, Anil Kanga, Vinay Shanker, Digvijay Singh, Poonam Gupta, Kiran Mokta, Vinita Sharma

**Affiliations:** 1 Department of Microbiology, Indira Gandhi Medical College, Shimla, Himachal Pradesh, India; 2 Department of Dermatology, Indira Gandhi Medical College, Shimla, Himachal Pradesh, India; Fundação Oswaldo Cruz, Brazil

## Abstract

Sporotrichosis is endemic in the Sub-Himalayan belt, which ranges from the northern to the north-eastern Indian subcontinent. Similar to many parts of the developing world, sporotrichosis is commonly recognized clinically in this region however consolidated epidemiological data is lacking. We report epidemiological, clinical and microbiological data from a hundred culture positive cases of sporotrichosis. Out of 305 clinically suspicious cases of sporotrichosis, a total of 100 isolates were identified as *Sporothrix schenckii* species complex (*S. schenckii*) on culture. Out of the culture proven cases 71% of the cases presented with lymphocutaneous type of lesions while 28% had fixed localized type and 1% had disseminated sporotrichosis. Presentation with lesions on hands was most frequently seen in 32% with arm (23%) and face (21%) in that sequence. The male to female ratio was 1∶1.27. Age ranged from 1 ½ years to 88 years. Mean age was 43.25 years. Disease was predominantly seen in the fourth to sixth decade of life with 58% cases between 31 and 60 years of age. Since the first report from the region there has been a steady rise in the number of cases of sporotrichosis. Seasonal trends reveal that most of the patients visited for consultation in the beginning of the year between March and April. This is the first study, from the most endemic region of the Sub-Himalayan belt, to delve into epidemiological and clinical details of such a large number of culture proven cases over a period of more than eighteen years which would help in the understanding of the local disease pattern of sporotrichosis.

## Author Summary

Sporotrichosis is a sub-acute or chronic granulomatous fungal infection involving mainly the skin and subcutaneous tissue with neighbouring lymphatics. It is caused by thermally dimorphic fungus, *Sporothrix schenckii*, which is prevalent worldwide. Consequent to trauma, the fungus establishes itself in skin and subcutaneous tissue of gardeners, forestry workers, farmers, carpenters, and others who are involved in outdoor activities. Sporotrichosis is the most frequently encountered sub-cutaneous mycosis in the sub-Himalayan belt and all previous reports are based on clinical review. It is imperative to understand the epidemiology of sporotrichosis as an accurate diagnosis, and prompt initiation of the appropriate treatment would prevent chronic debility. This is the first extensive compilation of culture proven cases from Himachal Pradesh in India. It thus justifies our interest in analysing data from our institute seen over 18 years and 7 months, with the objective to compare the demographic factors, briefing the clinical aspects and the culture characteristics of the isolates obtained from the clinical material.

## Introduction

Sporotrichosis is a sub-acute or chronic granulomatous mycotic infection involving primarily the skin and subcutaneous tissue with neighbouring lymphatics. It is caused by thermo-dimorphic fungus, *Sporothrix schenckii* which is prevalent worldwide growing on timber, sphagnum moss, plant detritus, soil, etc. Consequent to trauma, the fungus establishes itself in skin and subcutaneous tissue of gardeners, forestry workers, farmers, carpenters, and others who are involved in outdoor activities. *S. schenckii* is known to thrive at high humidity of 92–100% and a mean temperature ranging between 25°C–37°C [1], [2]. An uncommon mode of transmission includes bites and scratches from animals [3].

The disease characteristically occurs sporadically though large outbreaks have been reported. Since its first recognition by Benjamin Schenck in 1898 from Johns Hopkins Hospital in Baltimore, most cases have been reported from tropical and sub-tropical countries like Mexico in North America, Brazil and Peru in South America and Japan [1], [3], [4]. The first case in India was reported from Assam by Ghosh in 1932, followed by majority reports from Assam, West Bengal and Andhra Pradesh. Recent reports are from Chandigarh, Delhi, Sikkim, Uttar Pradesh, Punjab and Himachal Pradesh [1], [5]–[8].

Himachal is a small North Western hilly state of India with altitudes ranging between 350–6975 meters above the sea level [9]. The geo-climatic conditions are conducive for growth of *S. schenckii* as a saprophyte in the environment. Singh et al. described the first case of sporotrichosis from Himachal Pradesh in 1980 [6]. Since then, a large number of cases have been reported [5], [6], [10]–[13].

In 1908, Splendore identified asteroid bodies which are stellate forms consisting of a central spore surrounded by a mass of eosinophilic material in human tissue and named the fungus *S. asteroides*. Small oval cells resembling cigars in the tissue sections were described by de Beurmann and Gougerot [14]. Both these forms are rarely seen in direct microscopy or histopathological examination of the tissue. Serodiagnosis using complement fixation test, latex agglutination, precipitation test, fluorescence techniques or intradermal sporotrichin skin test, do not distinguish between cutaneous infections and uninfected individuals in endemic areas. Fungal culture remains the gold standard for establishing a definitive diagnosis of the disease [4], [15].

Sporotrichosis is the most frequently encountered sub-cutaneous mycosis in Himachal and all previous reports are based on clinical review. This is the first extensive compilation of culture proven cases from Himachal Pradesh in India. It thus justifies our interest in analysing data from our institute seen over 18 years and 7 months, with the objective to compare the demographic factors briefing the clinical aspects and the culture characteristics of the isolates obtained from the clinical material.

## Materials and Methods

A retrospective review of mycological records of culture proven sporotrichosis cases since January 1992 till July 2010 over a duration of 18 years and 7 months was conducted in our institute. Permission was taken from the Heads of Departments of Microbiology and Dermatology to access the records as was the study protocol. The demographic data of all patients' were noted. All the patients' data was anonymized. All samples were collected after taking written informed consent from the patients. This was noted onto the biopsy register maintained in the minor operation theatre in the Department of Dermatology where samples are collected. The skin biopsies, scrapings and pus samples submitted with the clinical possibility of sporotrichosis were processed in the routine mycology laboratory for direct microscopy and culture [2]. Demographic data of patients', whose diagnosis of sporotrichosis was established on the basis of fungal culture, were analysed using statistical methods (Chi-square method).

## Results

**Figure 1:**
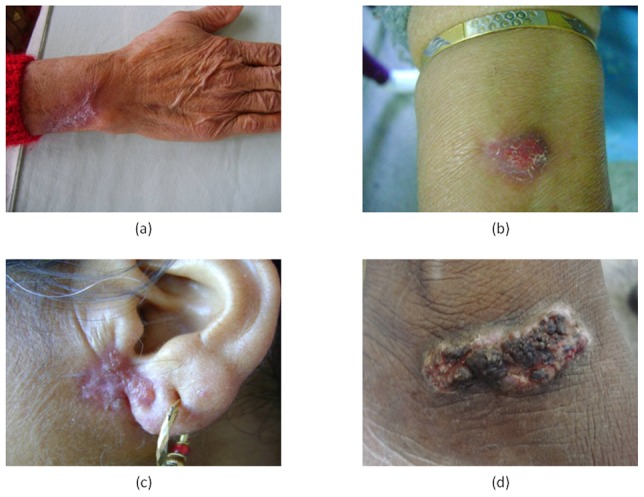
Fixed cutaneous type of lesions in cases of sporotrichosis. The figure shows lesions on the (a) wrist (b) arm (c) pinna and adjoining face and (d) dorsum of foot.

Total number of samples received in the mycology laboratory of the Microbiology department over duration of 18 years and 7 months was 2356. Out of these, 305(12.94%) samples were received with the clinical possibility of sporotrichosis. A total of 100 isolates were identified as *S. schenckii* on the basis of growth characteristics, macroscopic and microscopic morphology, and thermal dimorphism. Out of the hundred culture positive cases 71% cases presented with lymphocutaneous type, 28% had fixed localized lesions (Figure 1) and 1.0% of disseminated cutaneous form. The period prevalence of clinically suspected samples received for laboratory confirmation was 12.9%. However, etiology on basis of culture was established in 100 patients giving laboratory prevalence of 4.24%. Presentation with lesions on hands was most frequently seen in 32%, with arm (23%) and face (21%) in that sequence. Other presentations included 2 cases each with lesions on neck and abdomen and 1 case each with rare presentations on breast and disseminated sporotrichosis.

**Figure 2:**
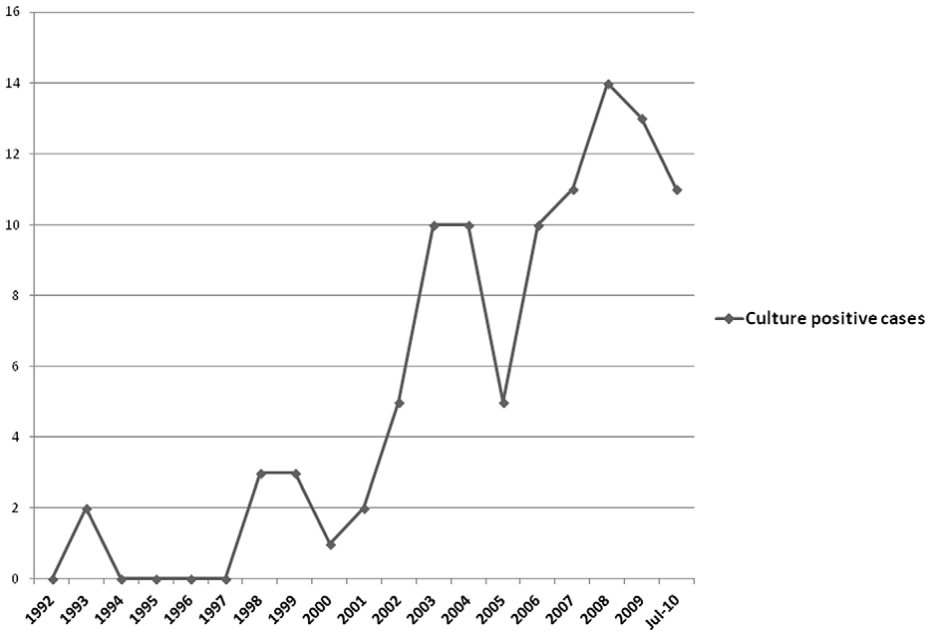
Year wise number of culture positive cases of *Sporothrix schenckii*.

**Figure 3:**
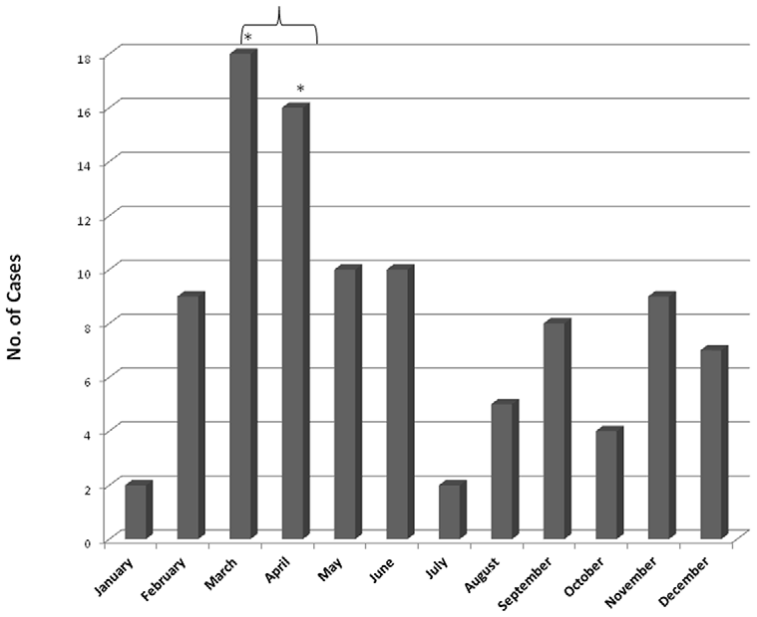
Month wise distribution of culture confirmed cases over the study period. A significantly higher number (*) of cases were seen in months of March and April (p = 0.0002).

Out of the 100 culture proven cases, 56 patients were females and 44 were males. The male to female ratio was 1∶1.27. Age ranged from 1 ½ years, the youngest child to 88 years, the oldest male patient. Mean age was 43.25 years. Seven children were <15 years old representing 4.21% of the study group. Although the disease was predominantly seen in the fourth to sixth decade of life, with 58% cases between 31 and 60 years of age, none of the age groups (decades) was significantly higher, [P value>0.05]. The average number of cases was 5.4 per year. To evaluate annual trends, the study period was compared in three parts. The number of culture proven cases in the first (1992–1997), second (1998–2003) and third (2004–July 2010) parts were 2, 24 and 74 respectively, giving a ratio of 1∶12∶37 (Figure 2). Seasonal trends reveal that most of the patients visited for consultation in the beginning of the year, with a highly significant number of cases presenting in March and April clubbed together [P value = 0.0002] with 63% of the visits occurring from February to June (Figure 3). In the present study, 90% of cases had agriculture as their main or spare-time job probably exposing them to contaminating injuries. Out of 100 cases, 61 were residents of rural areas subjecting them to environmental exposure. History of trauma with thorns, hay, wood splinters, needles, blades or injury due to fall was given in 47 patients out of which samples of 24 (51.06%) cases showed growth of *S. schenckii*.

Direct microscopy of one specimen revealed Asteroid bodies (1%) whereas no sample showed yeast cells. Only seven samples were assessed by PAS staining but results were inconclusive. Fungal cultures were observed twice a week up to 30 days and morphological appearance of characteristic growth ascertained using micro-slide culture technique. Growth characteristics showed few mm to one cm white yeast like colony in 3–6 days, yeast like, buff mycelial form in 7–10 days, buff mycelial form to black leathery growth in two weeks and black mycelial, leathery growth after 2–3 weeks. Retrospective data of growth characteristics of 79 isolates were retrievable. In this study, we observed that 90.6% (68/75) of the isolates showed growth within two weeks of inoculation and incubation at 25°C. Out of 100 isolates, 31/71(43.66%) of lymphocutaneous type showed culture positivity within the 2^nd^ week. While 11/28(39.28%) cases of fixed cutaneous form showed growth within 14 days. Dimorphism was demonstrated by yeast conversion on brain heart infusion blood agar after incubation at 37°C and 91.30% (63/69) showed yeast conversion within two weeks (Table 1). Patients were treated with saturated solution of potassium iodide (SSKI) therapy. In cases of intolerable side-effects due to SSKI, itraconazole and fluconazole were the alternate anti-fungal agents.

**Table 1:** Characteristics of growth in isolates of *Sporothrix schenckii*.

Growth in days	No. of samples isolated at 25°C	No. of samples isolated at 37°C	Yeast Conversion at 37°C
**3–6 days**	4	0	5
**7–10 days**	43	0	32
**11–14 days**	21	1	26
**>15 days**	7	3	6
**TOTAL**	75	4	69

## Discussion

Himachal Pradesh is a small hill state in North-West India bounded between 30°22′ to 33°12′ North latitude and 75°47′ to 79°04′ East longitude lying entirely in the Western Himalayas. The whole terrain is mountainous with altitude varying from 350 m to 6975 m above the mean sea level. Himachal Pradesh encompasses wide range of agro-climatic conditions conducive to horticulture and agriculture. These are integral to the economy and sustainability of people with 90% of the population dependent on this primary sector. Out of a total of 60,77,900 population, rural community constitutes 54,82,319 and 8,63,000 population are primarily farmers by occupation [9].

*S. schenckii* is a thermally dimorphic fungus existing as a mould at 25°C and as yeast in host tissue at temperature of 37°C [2]. It is widely distributed in nature as a common soil saprophyte occurring on decaying wood, corn stalks and sphagnum moss as has been reported by Mehta et al. from Himachal Pradesh [12]. Professionals involved in agricultural activities like gardeners, farmers, orchardists, florists, foresters or persons whose occupational or recreational activities bring them in contact with ecotypes favourable with saprophytic growth of the fungus and are associated with traumatic contact with plant material and soil are at greater risk of acquiring sporotrichosis.

Age, sex and race have no epidemiological significance in disease causation. Different studies report male predominance [1], [16] or females [1], [6], [8], [17], [18] being more frequently affected. The present study shows a female preponderance (M∶F = 1∶1.27). The disease is related to outdoor practices. In our state, both men and women cultivate and plough the land and collect firewood and fodder from forests along with rearing of livestock. Thus, women too are at high risk of acquiring the disease accounting for a significant number of cases. Previous reports document sporotrichosis in infants as young as 87 days in a study and16 months in another and persons as old as 71 and 87 years [17], [19].The age ranged from 18 months to 88 years in the present study. A rising incidence was observed with increasing age peaking at 4^th^ and 5^th^ decades and declining thereafter. These trends correlate with the active years of the rural inhabitants when they are maximally exposed to occupational trauma and subsequent infection. Children less than 15 years accounted for a meagre 8% which is in sharp contrast with the study from Peru where 60% infections were reported in this age range [3]. The regional variations in the age and sex distribution of cases are usually attributable to different exposure conditions related to economy and literacy. Our findings are however corroborative with other studies [4] where 15% children were <10 years. Previous reports from Himachal reveal similar occurrence [6].

Humidity and rainfall are prime environmental factors predisposing to disease. The temperature between 25°C and 37°C and high humidity of 92–100% helps growth of *S. schenckii* in the environment [2]. The lower hills of the state known as the Sub-Himalayas have annual rainfall of 1500 mm to 1800 mm with average rainfall of 152 cm accounting for high humidity. Widespread rain occurs from late June to September and part of the state experiences semi-tropical to semi-arctic conditions. In paradox to the expected, we observed 63% of hospital visits between February and June which is the cooler, drier part of the year with sporadic rains and minimum cases during fall and peak winter. Ghosh et al. [5] have reported rain and humidity as two significant factors for sporotrichosis. Similarly, in South Africa, the temperature of 26°C–27°C and 92–100% humidity were known to favour occurrence of sporotrichosis. Contrary to this, in Mexico, greater frequency of infection has been reported during the dry and cooler part of the year similar to our observations [5]. With our experience of this endemic region, we comprehend that though the villagers acquire infection during rainy season, they usually visit peripheral health facilities close at hand where diagnostic modalities for confirmation of fungal infections are not available. Keeping in view infections other than sporotrichosis with similar looking lesions, the therapy instituted usually does not cover fungal infections. Patients do not respond to the treatment at these centres and are referred to tertiary health care hospitals, to a dermatologist after an average duration of disease of 6 to 10 months. The suitability of time when the tilling activities in the land holdings are minimum labour intensive is another local factor accounting for regional trends.

The disease prevalence has perceptibly increased over the years. Perhaps, the increased education, awareness and the literacy rate of 76.05% existent in Himachal Pradesh [9] makes the people realise the need to visit the hospital for ailments. Furthermore, the disease is frequently suspected by the treating physician at the tertiary health care facility thereby accounting for rising incidence. There was a significant rise in number of culture confirmed cases in 2003 and again in 2010 which cannot be accounted for by the above factors alone. The reasons cannot be elucidated in absence of more elaborate epidemiological studies and further identification of the isolates. To validate hypothesis like increase in environmental distribution of *S. schenckii* or change in virulence of the agent, extensive studies need be done.

Clinical cases of sporotrichosis have been classified into lymphangitic or lymphocutaneous lesions, localized or fixed type, multifocal or disseminated and extracutaneous categories by Sampaio and Lacaz [20]. The classic form is lymphocutaneous accounting for nearly 70% of cutaneous sporotrichosis cases [2]. Our findings corroborated fully with this as we also reported 71% of the cases to have lymphocutaneous lesions. Lesions on hands were the most frequent presentation followed by arm, face, legs and foot in that order [2]. We came across rare sites like lesions on breast, ear lobe and disseminated presentations during our study.

The lesions at inoculation site can be multiple, may remain as such or nodules appear along the lymphatics. Hematogeneous spread or multiple traumatic implantations of the fungus is perhaps responsible for cutaneous dissemination and may even be seen in individuals apparently without any predisposing factors for immunosuppression [2]. Extracutaneous forms like pulmonary, osteoarticular, ocular, central nervous system disease, etc., though reported both in immunocompetent and immunosuppressed cases have not been encountered during the study period. It has also been experienced that only 5.06% (4/79) of the isolates showed growth at 37°C thereby implying that the local strain in Himachal probably is unable to thrive at internal body temperature thus unlikely to cause extracutaneous disease (Table 1).

Histopathology is usually non-specific and rarely diagnostic as the cigar-shaped, budding yeast-like cells are scanty in the pus or tissue samples, rarely demonstrable and invariably non-contributory in confirmation of diagnosis [6], [19]. Asteroid bodies (Splendore-Hoeppli phenomenon) in pus were originally described by Splendore in 1908 [16]. They consist of a central round cell of 5–13 microns, occasionally budding, surrounded by club-shaped multiple hyaline projections. Its demonstration serves only as an adjuvant diagnostic test as eumycotic mycetoma, actinomycosis and granules associated with *Pseudomonas aeruginosa* may show presence of Asteroid structures. Researchers have variously reported presence of Asteroid bodies as 40% to 85.7%; however, many researchers point out the rarity of these forms in human lesions [16]. In the present study, direct microscopy was performed in 58 samples and Asteroid bodies were revealed in a solitary case (1%) whereas yeast- like cells were not observed in any sample. Special stains like PAS are usually not helpful in recognition of sporotrichosis due to paucity of yeast in tissue and none of the specimens stained by PAS contributed in diagnosis in our study. Lack of experience in visualizing fungal forms in these stains together with rarity of yeast cells in specimens are reasons apprehended for inability to recognise fungal elements.

The chances of isolating *S. schenckii* from sporotrichosis cases are very good [2] giving a confirmed diagnosis in 2 weeks as seen in our study. The culture is characterised by initial white yeast- like growth appearing within 10 days followed by buff coloured mycelial forms in another 4–5 days. The colour changes to greyish-black with a leathery to velvety texture with occasional aerial mycelium over 2 weeks. *S. schenckii* usually does not grow straight from the human specimen, but after growing at 25°C, there is a conversion at 37°C.

In the present account, we observed that 81% (64/79) of the isolates showed growth in the second week after inoculation and incubation at 25°C. Out of 100 isolates, 71 were lymphocutaneous of which 31 (43.66%) showed culture positivity within the 2^nd^ week. While out of the 28 cases of fixed cutaneous form of sporotrichosis, only 11 (39.28%) showed growth within 14 days. This curious aspect revealed while studying growth of isolates is corroborated by facts cited previously in literature [2], [13], [21].

Other immunological techniques having little diagnostic significance in healthy people and are not recommended in endemic areas thus were not undertaken. Patients were treated with saturated solution of potassium iodide (SSKI) therapy as it is cheap, effective and gives consistently effective results in a dermatologist's experience. Favourable response within 2 weeks in most cases with healing in 4–8 weeks or up to 32 weeks was observed. To achieve mycological clearance, therapy may be continued for another 4–8 weeks. In cases of intolerable side-effects due to SSKI, itraconazole and fluconazole were the alternate anti-fungal agents used and encouraging results were seen as also documented by other workers [6].

Our present observation demonstrates and emphasises the importance of culture of samples from suspected cases. Fungal culture is gold standard test of great diagnostic significance especially in cases of clinical ambiguity where lesions are not differentiable from lupus vulgaris, cutaneous leishmaniasis, nocardial infections, chromoblastomycosis, syphilis, tuberculosis verrucosa cutis, foreign body granuloma, etc. Culture is undoubtedly the modality of choice for diagnosis, especially because sometimes histopathology gives inconclusive results and usually direct microscopy is non-contributory. We also conclude that there is a significant rise in incidence of sporotrichosis in the sub-Himalayan region.
